# Supplementary material for: Lactiplantibacillus plantarum Postbiotics Suppress Salmonella Infection via Modulating Bacterial Pathogenicity, Autophagy and Inflammasome in Mice
Source: Animals (Basel). 2023 Oct 14;13(20):3215. doi: 10.3390/ani13203215 (PMC10603688; doi:10.3390/ani13203215)
Supplement: Supplementary file 1 [file animals-13-03215-s001.zip › Figure S4 Original bands for figure 6b.pdf]

p-ULK1-1

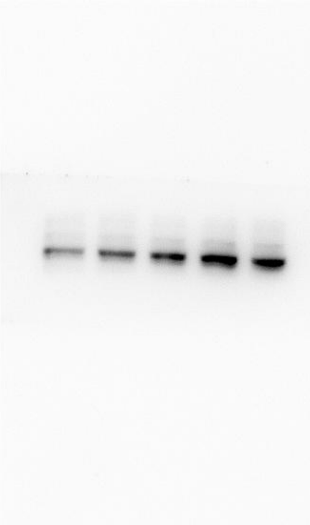

p-ULK1-2

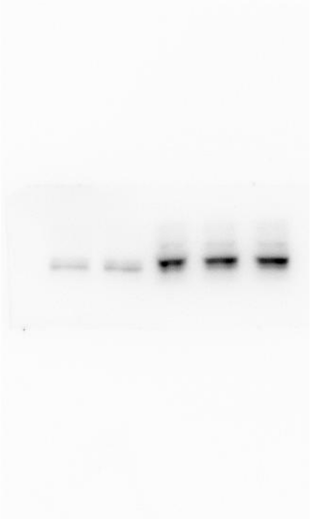

p-ULK1-3

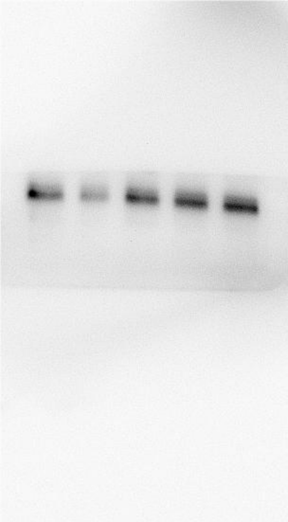

ULK1-1

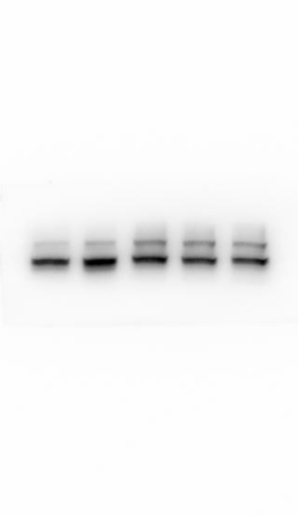

ULK1-2

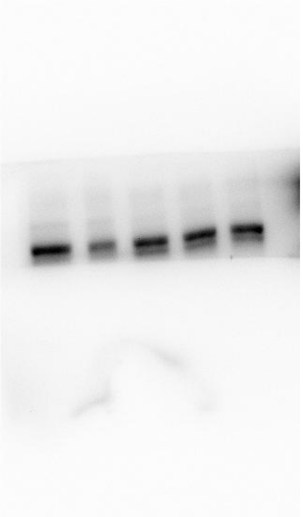

ULK1-3

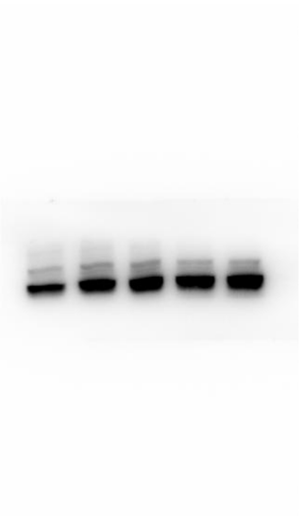

TAK1-1

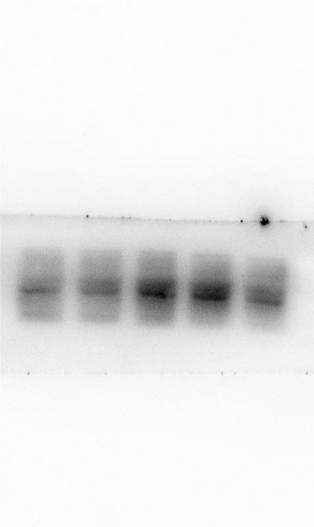

TAK1-2

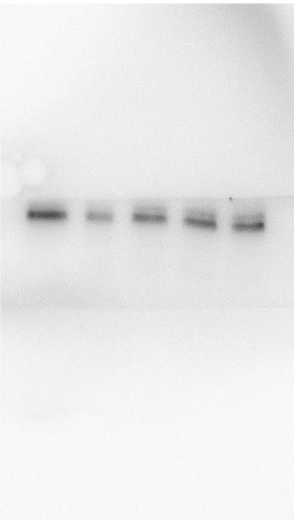

TAK1-3

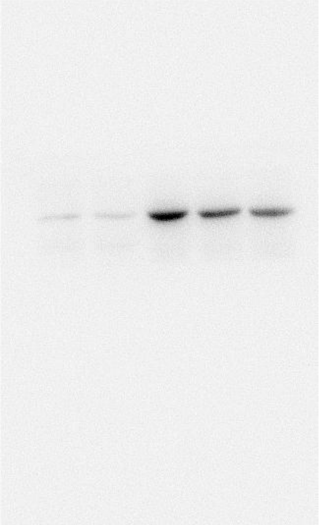

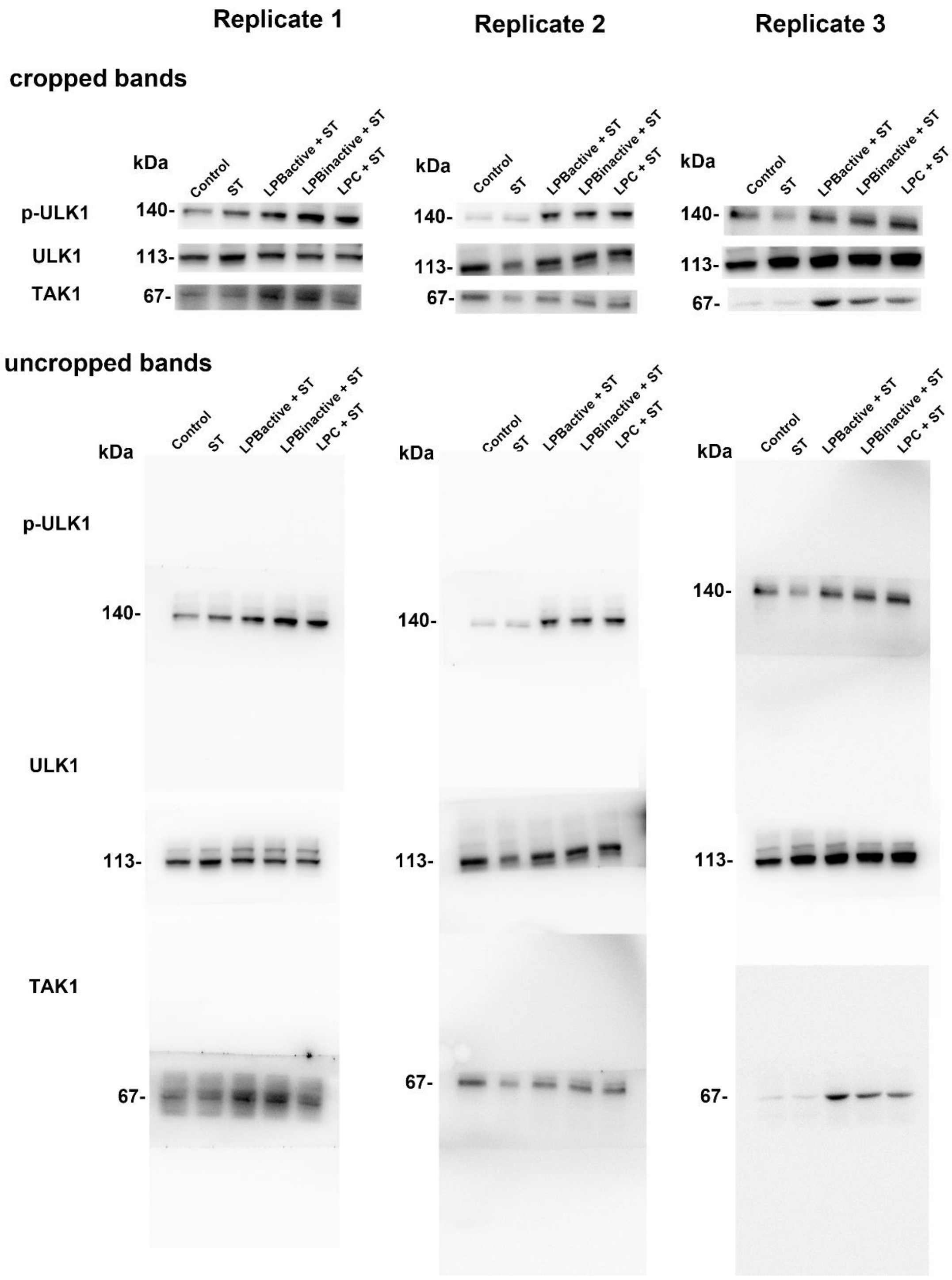

Replicate 1
Replicate 2
Replicate 3
cropped bands
kDa
Control
ST
LPBactive + ST
LPBinactive + ST
LPC + ST
p-ULK1
ULK1
TAK1
140-
113-
67-
uncropped bands

p-AMPK-1

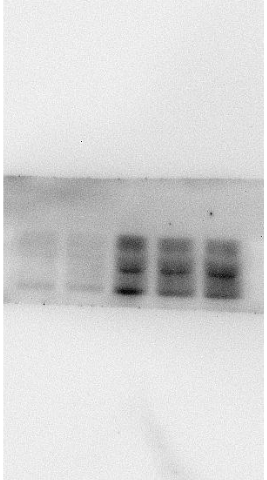

p-AMPK-2

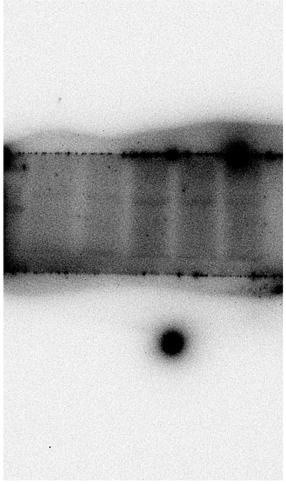

p-AMPK-3

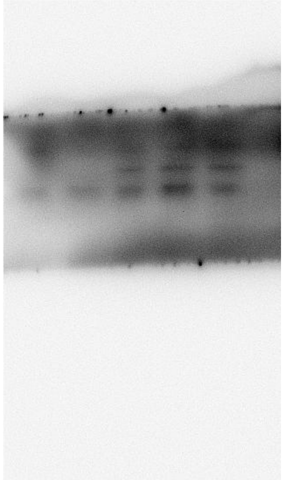

AMPK-1

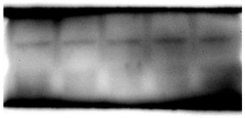

AMPK-2

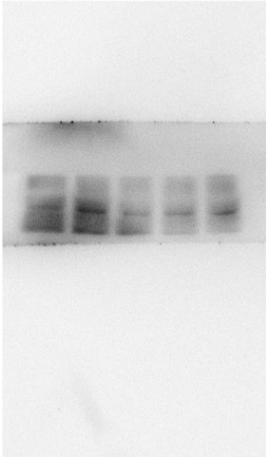

AMPK-3

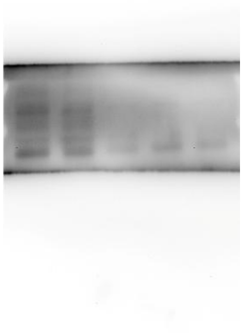

β-actin-1

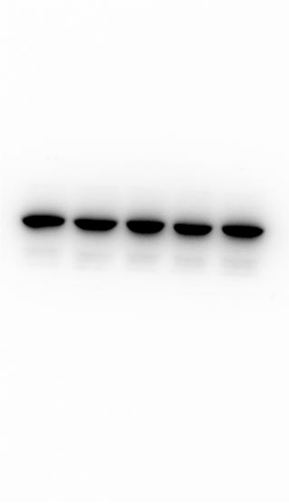

β-actin-2

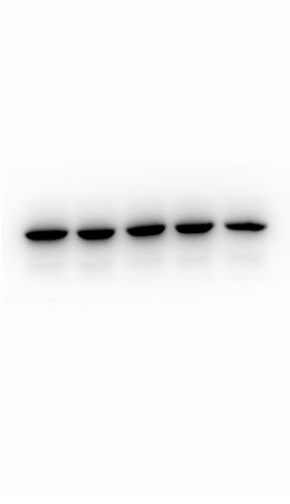

β-actin-3

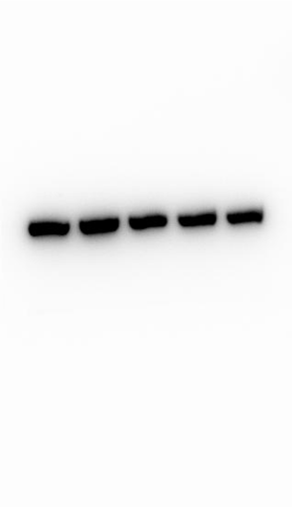

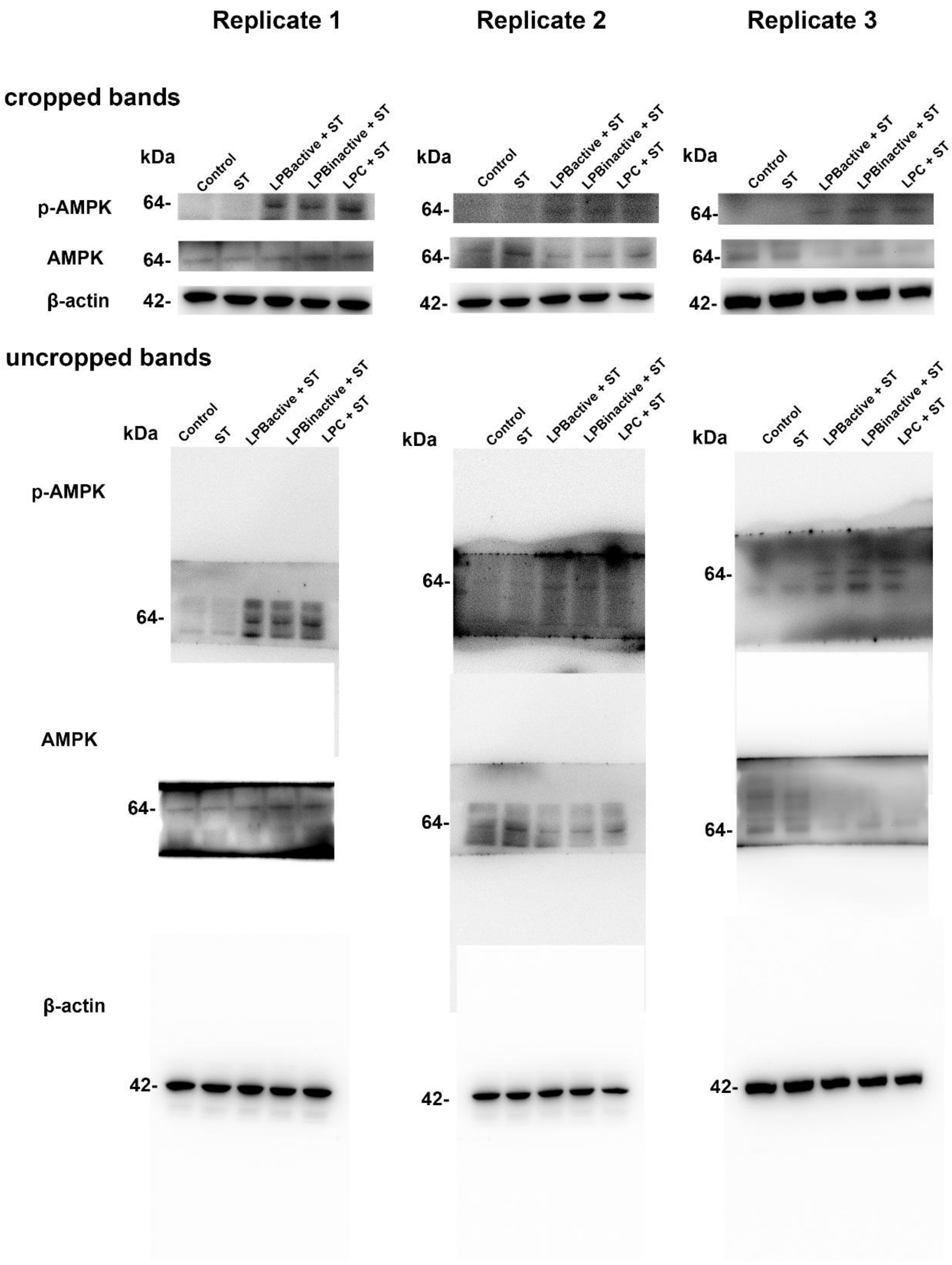
Replicate 1
Replicate 2
Replicate 3
cropped bands
kDa
Control
ST
LPBactive + ST
LPBinactive + ST
LPC + ST
p-AMPK
64-
AMPK
64-
β-actin
42-
uncropped bands
p-AMPK
64-
AMPK
64-
β-actin
42-
