# Supplementary figures and images for: Lactiplantibacillus plantarum Postbiotics Suppress Salmonella Infection via Modulating Bacterial Pathogenicity, Autophagy and Inflammasome in Mice
Source: Animals (Basel). 2023 Oct 14;13(20):3215. doi: 10.3390/ani13203215 (PMC10603688; doi:10.3390/ani13203215)

Claudin-1-1

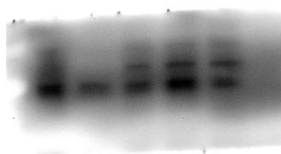

Claudin-1-2

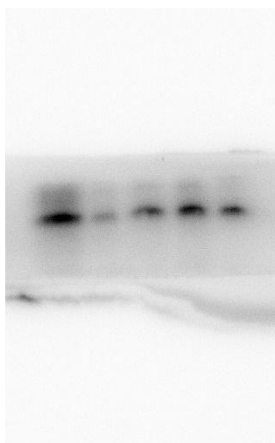

Claudin-1-3

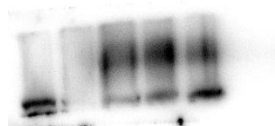

Occludin-1

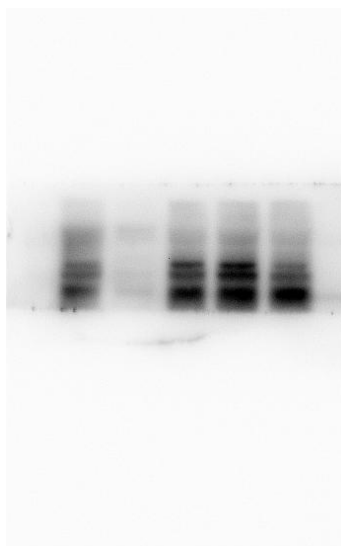

Occludin-2

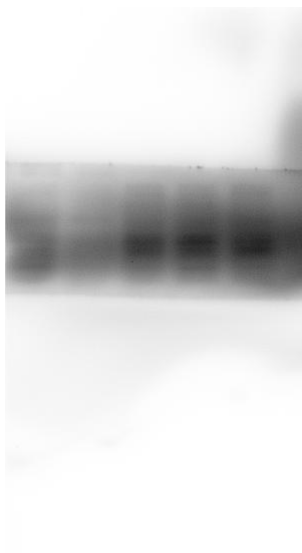

Occludin-3

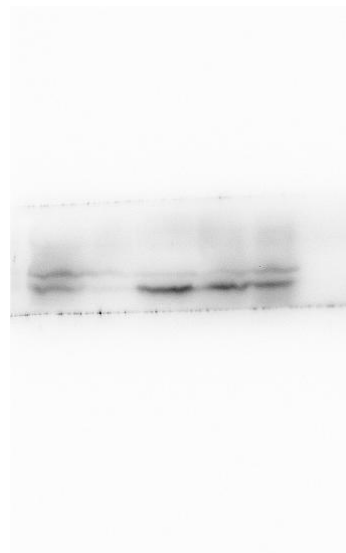

$\beta$ -actin-1

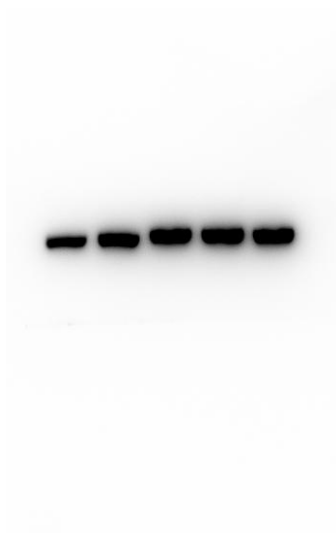

$\beta$ -actin-2

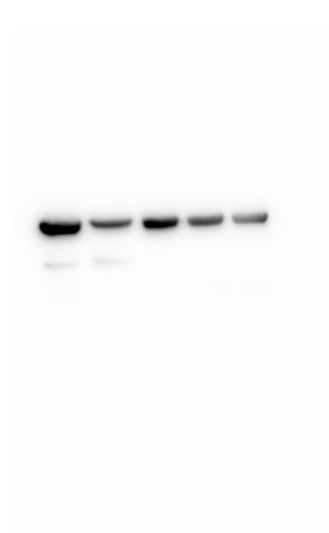

$\beta$ -actin-3

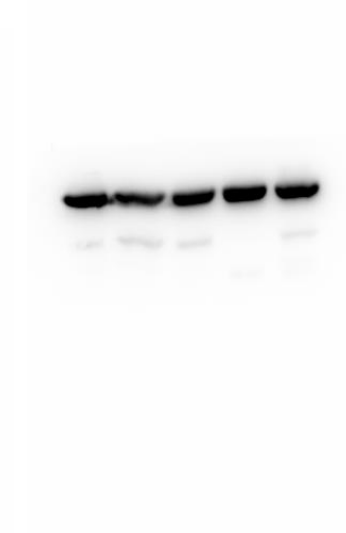

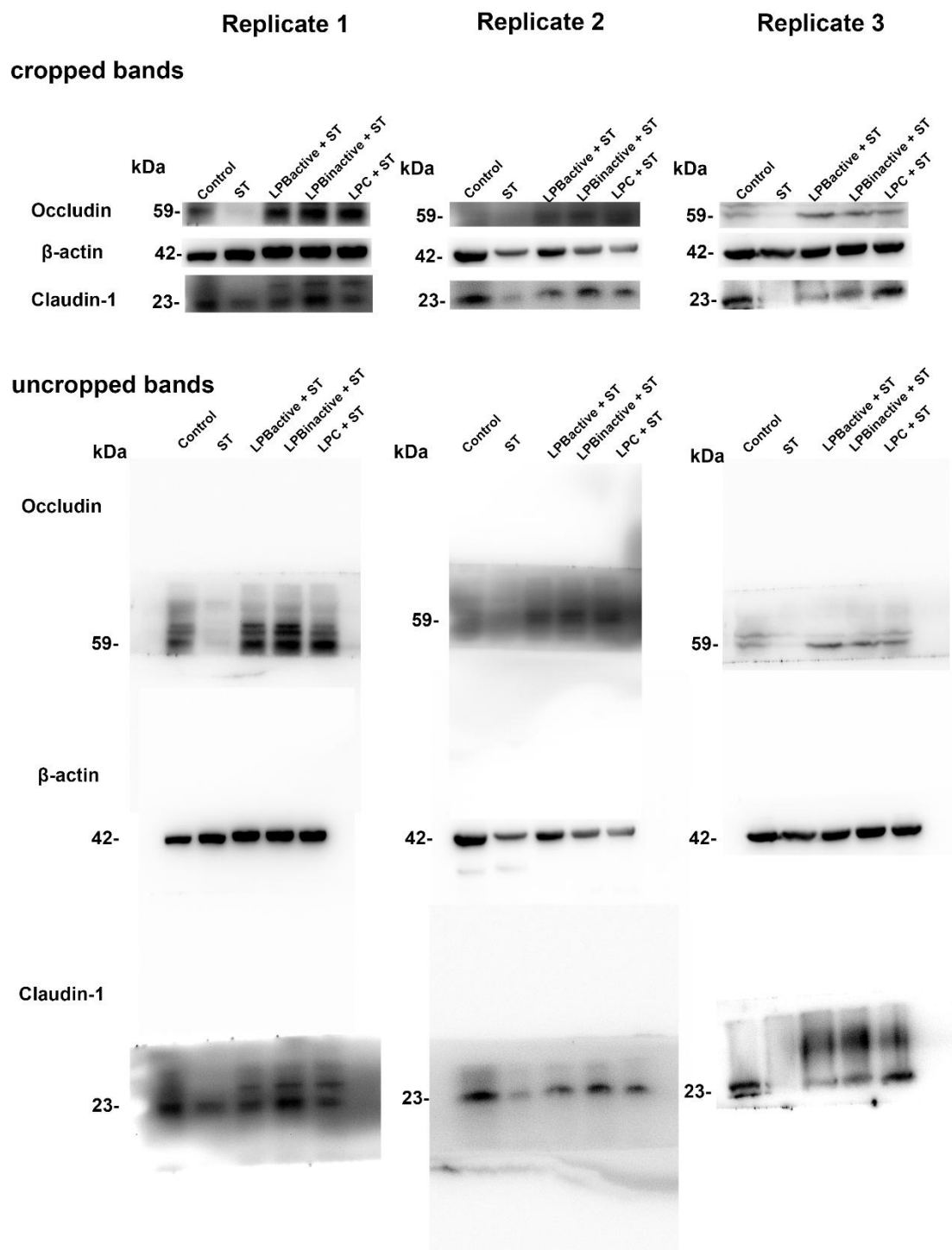

Supplement: Supplementary file 1 [file animals-13-03215-s001.zip › Figure S1 Original bands for figure 3c.pdf]

Caspase-1-1

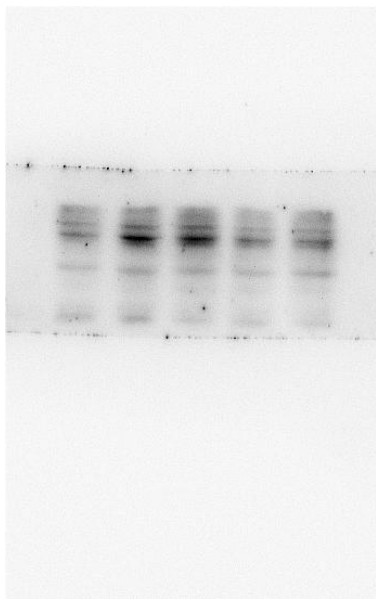

Caspase-1-2

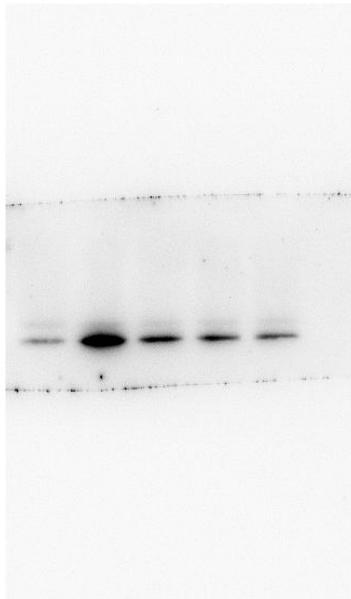

Caspase-1-3

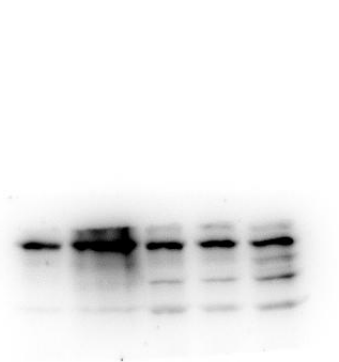

NLRP3-1

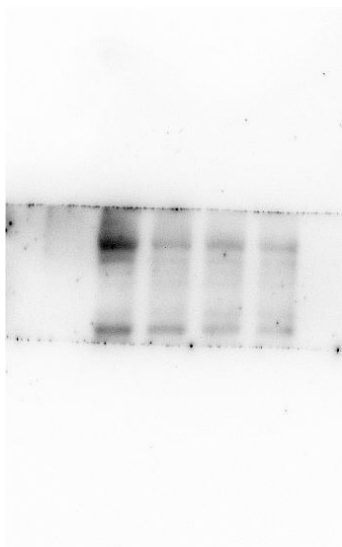

NLRP3-2

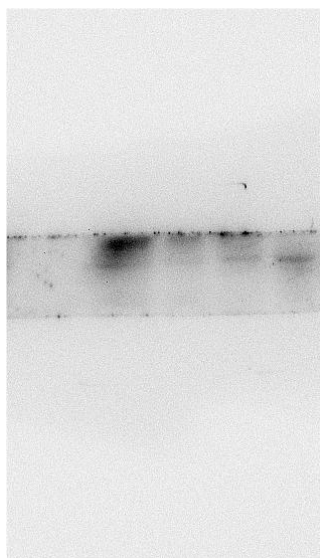

NLRP3-3

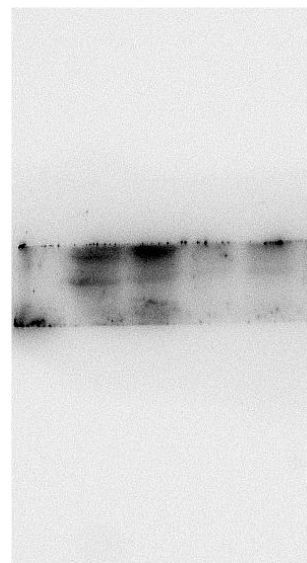

$\beta$ -actin-1

$\beta$ -actin-2

$\beta$ -actin-3

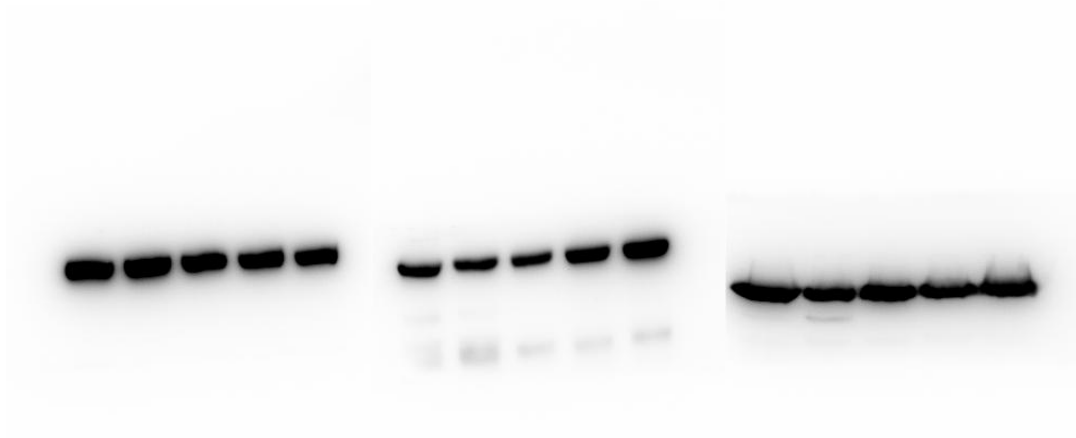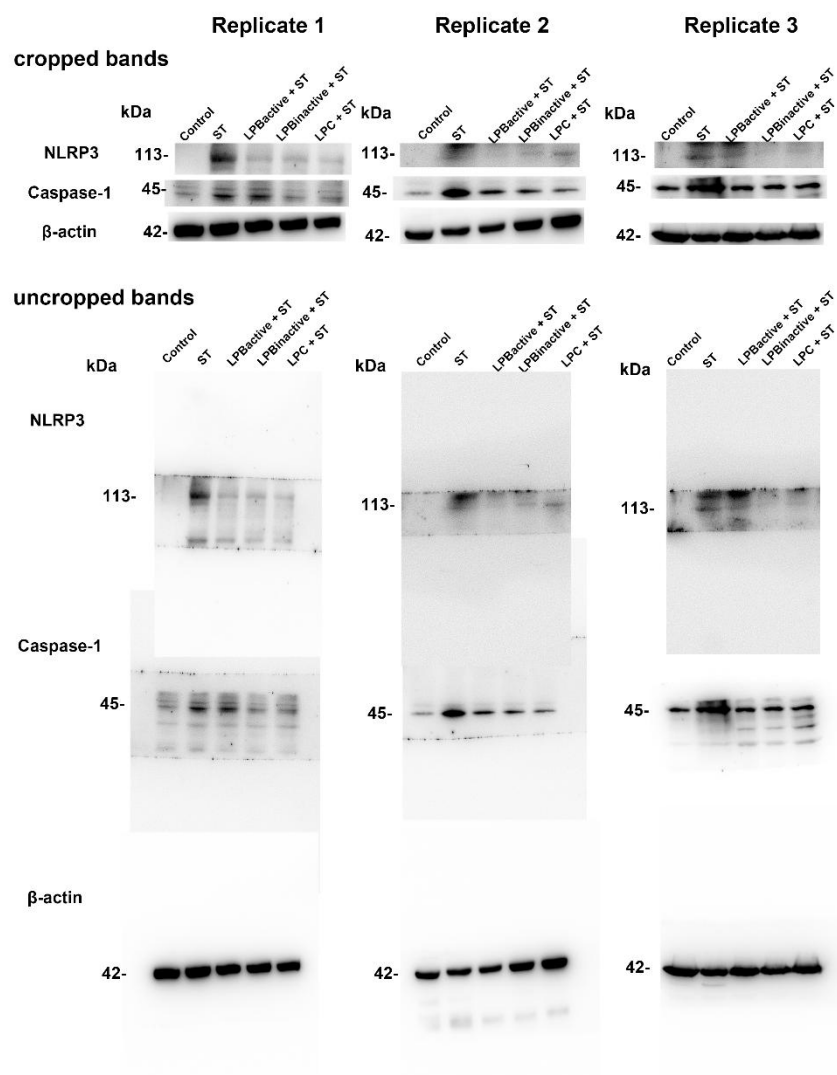

Supplement: Supplementary file 1 [file animals-13-03215-s001.zip › Figure S2 Original bands for figure 5b.pdf]

**Beclin1-1**

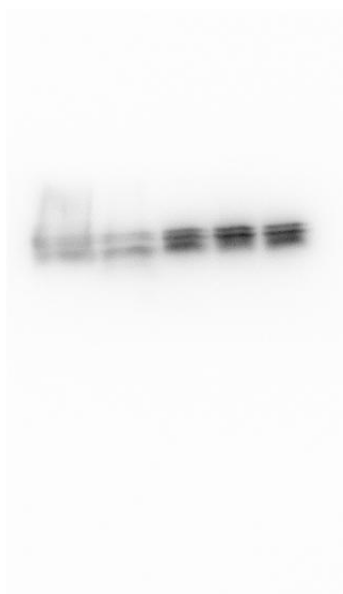

**Beclin1-2**

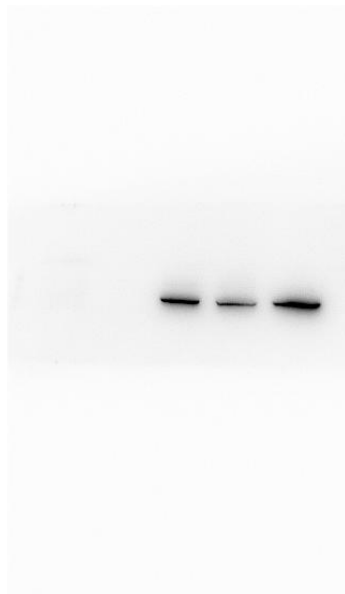

**Beclin1-3**

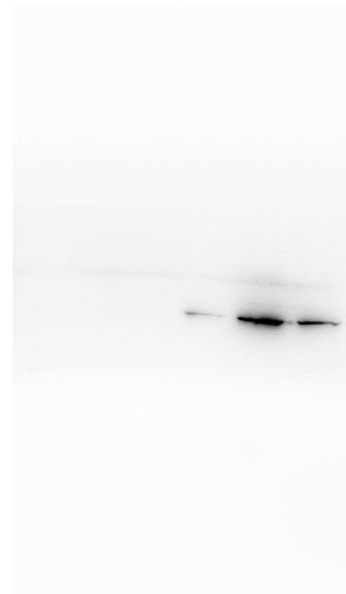

**LC3 -1**

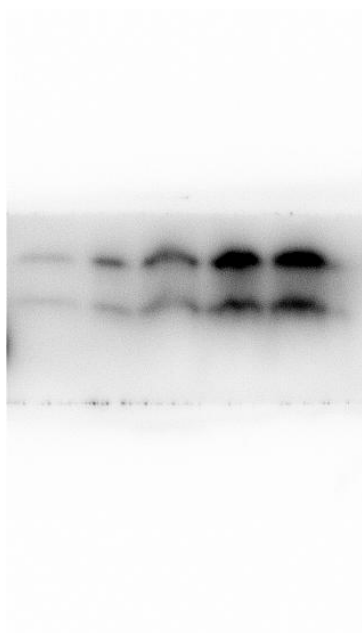

**LC3 -2**

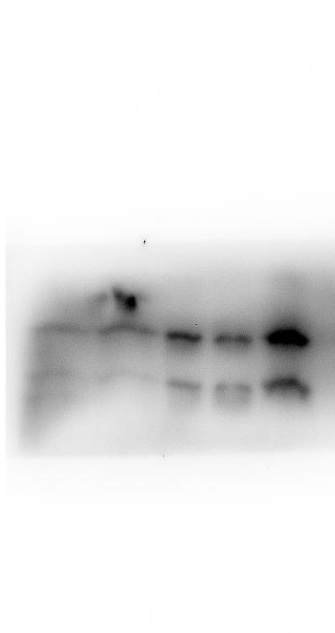

**LC3 -3**

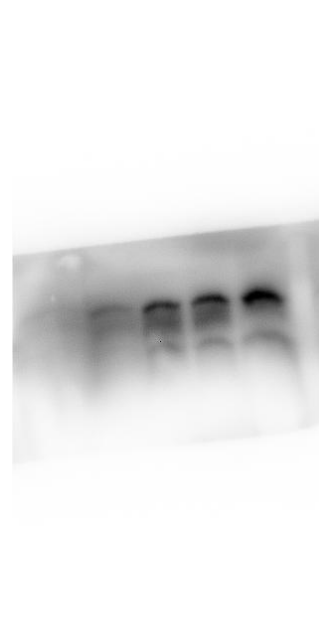

P62-1

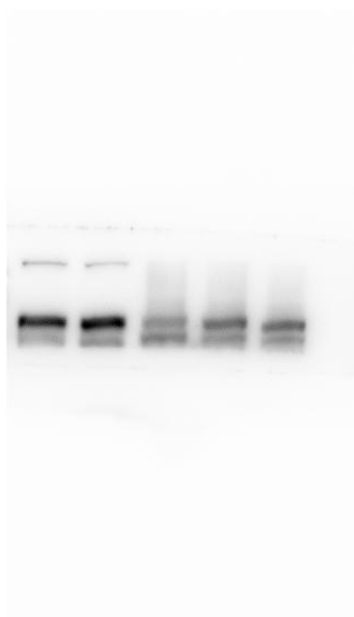

p62-2

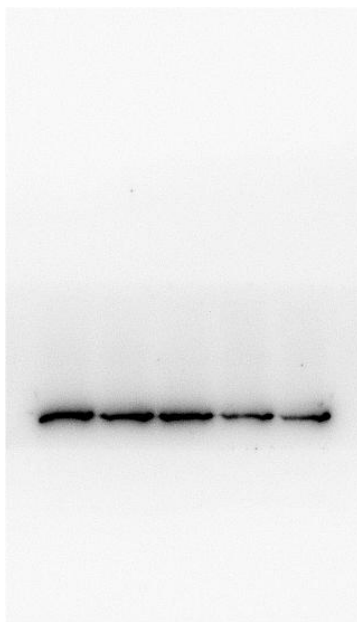

p62-3

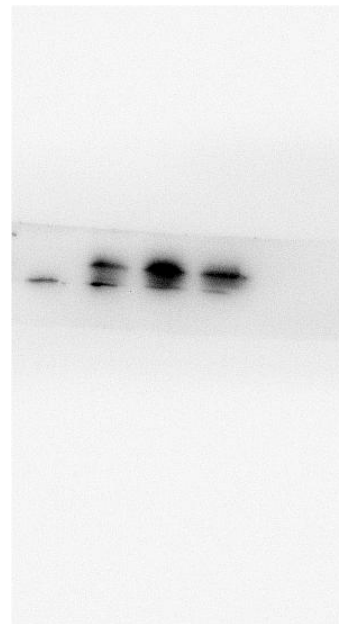

$\beta$ -actin-1

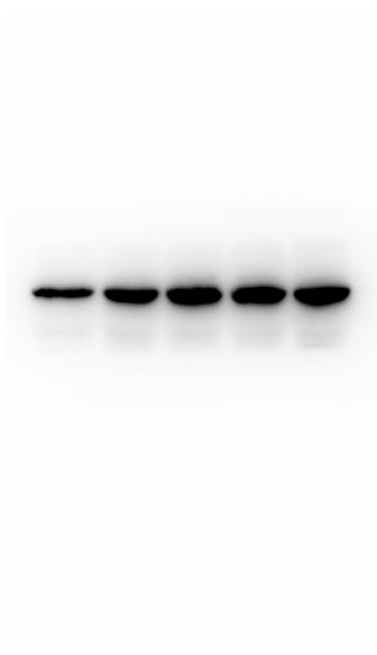

$\beta$ -actin-2

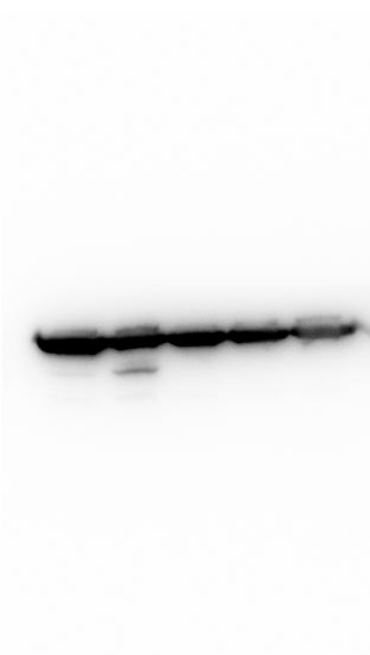

$\beta$ -actin-3

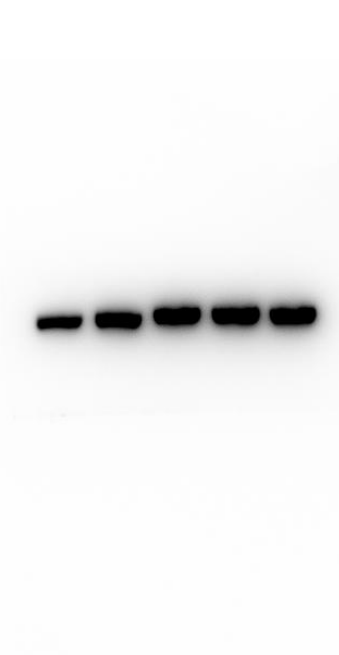

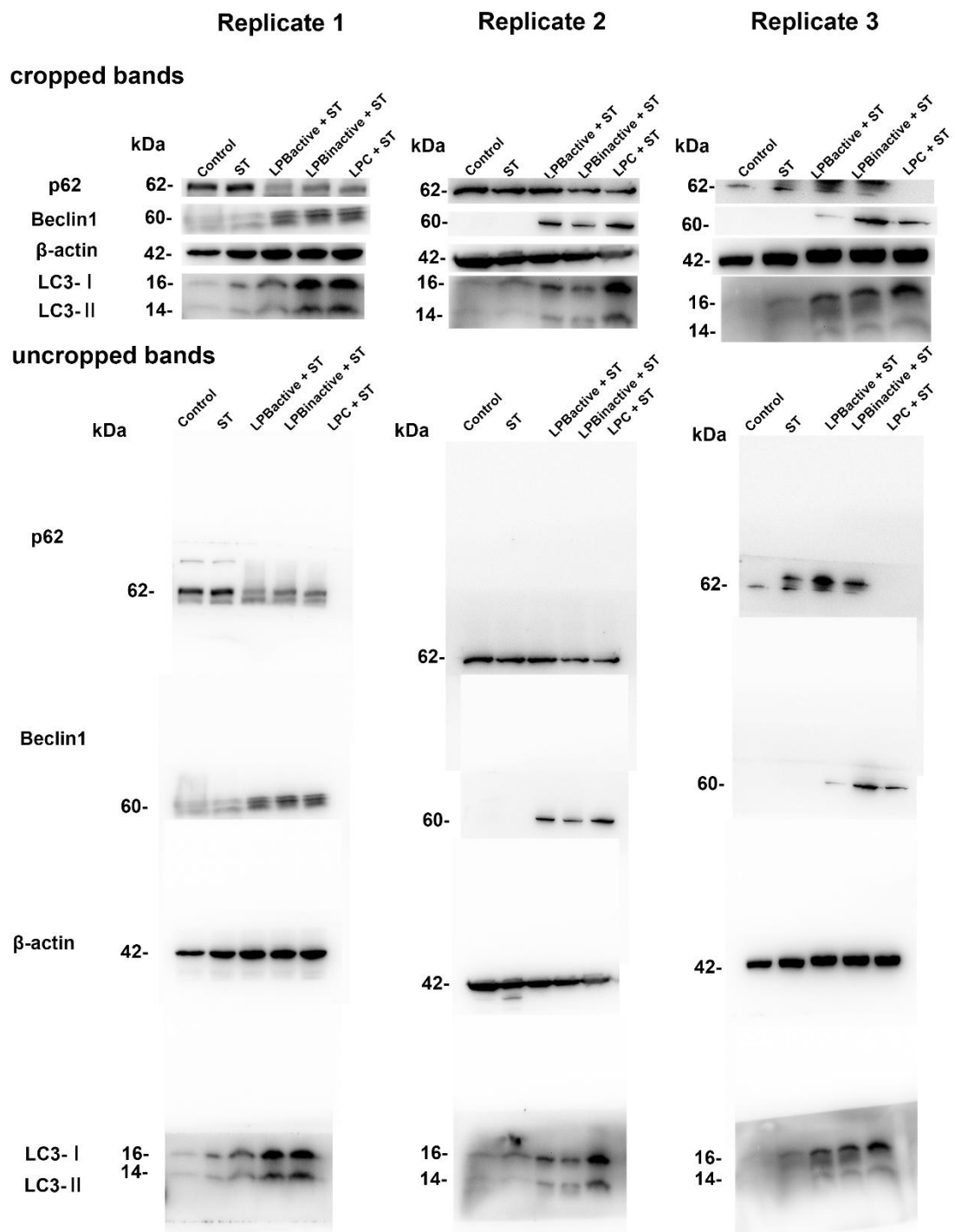

Supplement: Supplementary file 1 [file animals-13-03215-s001.zip › Figure S3 Original bands for figure 6a.pdf]
